# Ultramarathon Evaluation above 180 km in relation to Peak Age and Performance

**DOI:** 10.1155/2022/1036775

**Published:** 2022-08-26

**Authors:** Raphael Fabricio de Souza, Mirella Martineli Sousa Santos, Mabliny Thuany, Devisson dos Santos Silva, Micael Deivison de Jesus Alves, Davi Pereira Monte Oliveira, Beat Knechtle, Ana Filipa Silva, Filipe Manuel Clemente, Hadi Nobari, Felipe J. Aidar, Georgian Badicu, Stefania Cataldi, Gianpiero Greco

**Affiliations:** ^1^Department of Physical Education, Federal University of Sergipe (UFS), São Cristóvão, Brazil; ^2^Graduate Program of Physical Education, Federal University of Sergipe (UFS), São Cristóvão, Sergipe, Brazil; ^3^Group of Studies and Research of Performance, Sport, Health and Paralympic Sports-GEPEPS, Federal University of Sergipe, UFS, Sergipe, Brazil; ^4^Centre of Research, Education, Innovation and Intervention in Sport (CIFI2D), Faculty of Sports, University of Porto, 4200-450 Porto, Portugal; ^5^Institute of Primary Care, University of Zurich, 8091 Zurich, Switzerland; ^6^Escola Superior Desporto e Lazer, Instituto Politécnico de Viana do Castelo, Rua Escola Industrial e Comercial de Nun'Álvares, 4900-347 Viana do Castelo, Portugal; ^7^Research Center in Sports Performance, Recreation, Innovation and Technology (SPRINT), 4960-320 Melgaço, Portugal; ^8^Instituto de Telecomunicações, Delegação da Covilhã, 1049-001 Lisboa, Portugal; ^9^Department of Physiology, School of Sport Sciences, University of Extremadura, 10003 Cáceres, Spain; ^10^Department of Exercise Physiology, Faculty of Educational Sciences and Psychology, University of Mohaghegh Ardabili, Ardabil 56199-11367, Iran; ^11^Department of Physical Education and Special Motricity, Faculty of Physical Education and Mountain Sports, Transilvania University of Brasov, 500068 Brasov, Romania; ^12^Department of Basic Medical Sciences, Neuroscience and Sense Organs, University of Study of Bari, 70124 Bari, Italy

## Abstract

**Background:**

Ultramarathons with distances over 180 km might lead to different results regarding participation, performance, and age compared to shorter runs of 50 and 100 km.

**Objective:**

To evaluate ultramarathons with distances above 180 km in relation to runners' peak age and performance.

**Method:**

s. Verification of the quantity of competitions in runs over 180 km by continents in the period 2000 to 2020 and evaluation of the individual results of 13,300 athletes after 2010.

**Results:**

Europe stood out with the largest number of organized events, followed by Asia and North America. The age peak performance (PP) in men and women averaged 45 years old with relationship between sex × years (*F* = 3.612, *p* < 0.001; *η*^2^ = 0.003). Men accounted for more than 80% of the runners and showed a reduction in PP from 2015 onwards (*p* < 0.001). Competitions between 180 and 240 km were the most frequent, particularly after 2016, surpassing the number of marathons over 360 km (*p* < 0.001). Men and women showed higher velocity in distances (*p* < 0.001) from 180 to 240 km when compared to 241 to 300 k m, 301 to 360 km, and >360 km courses.

**Conclusions:**

The decade between 2010 and 2020 showed an increase in the number of Ultramarathon running events. Europe had the highest number. Women had low participation. Performance progression fell, a fact associated with an increase in the number of participants and not specifically related to a decline in athletic performance over the years.

## 1. Introduction

In recent years, there have been a consolidation and increase in the number of Ultramarathon (UMs) events around the world [1]. Previous research has been carried out in order to understand the profile of runners (i.e., sex, age, and economic status), age peak, and performance progression (PP), especially in 50 km and 100 km runs [2]. In summary, these studies have shown that the runners are male, aged over 35 years, and have undergone systematic training [3].

Generally, ultramarathoners were the oldest, compared to marathon and half-marathon runners [4]. When analyzing the performance of 2,067 100 km runners around the world during the past 59 years, an increase in the number of participants over 60 years was identified [5]. For 100 km, runners between 45 and 49 were the most successful athletes [2]. In the 161 km marathon, the age between 40 and 44 were the years with the highest number of successful finishers. Furthermore, it has been found that athletes over 70 years of age have improved running velocity every decade [1].

Previous studies have indicated that age [4], anthropometric characteristics [6, 7], and training experience [8] were associated with successful ultramarathon performance, especially in distances up to 100 km in the first days of the UMS. However, currently, the participation in challenges over 180 km is advancing. These competitions are considered extreme events exceeding 1000 km, making them a superior challenge, which presents the athletes with a series of adversities (i.e., extreme weather conditions and nutritional care) [9]. Thus, an understanding of the profile of athletes is important to guide their training and preparation for long-term performance.

An analysis of an ultramarathon over 180 km might lead to different results regarding participation and performance for both elite and aged group runners compared to an event covering distances such as 50 and 100 km. Research on variations in sex differences by age group would be expected to provide insights into differences in biological mechanisms of aging (e.g., hormonal changes) between women and men runners [10]. Further, UMs are quite different than shorter distances; there is great interest in knowing peculiarities in capabilities owing to the low [9]. An analysis of this difference in velocity might affect performance level.

This analysis is essential in strategic sports planning as it directs and contributes to the planning of the evolutionary phases up to the peak of physical development and potentiates victory in professional-level sporting events [4]. Despite the relevance of the studies already existing, there is a lack of data about the participation and performances in the UMs longer than 100 km. Our hypothesis was that the runners were more experienced, had higher PP, and ran at a reduced speed, given the increased distance. The objective of the present study was to evaluate ultramarathons above 180 km in relation to peak age and performance of the participants.

## 2. Materials and Methods

The present study presents a retrospective cohort design. A total of 1,202 worldwide ultramarathon events taking place between 2000 and 2020 were analyzed, with distances ≥ 180 km. All data used were downloaded manually from the DUV website (Deutsche Ultramarathon Vereinigung; https://statistik.d-u-v.org/) during February/2021.

A first analysis of the frequency of races in the period 2000 to 2020 was carried out. Based on these data, a second analysis was carried out for the years 2010 to 2020 (identified as an expressive period of the quantity of organized races), on the individual results of 13,300 athletes (11,646 men and 1,654 women), in the top 20 ultramarathoners. For analyzing average velocity over these years, both top 10 and top 20 ultramarathoners were also analyzed. The variables analyzed were as follows: age, sex, speed, distance, performance, and nationality. The athlete's age was computed taking into account only the year of birth; then, this information was used to determine the age of peak performance, taking into account previous studies [4].

### 2.1. Statistical Analysis

Descriptive analyses were performed to present the main characteristics of the events (number of events, distance of competition, number of finalists, and continental predominance) and runners (year of birth and sex). Data characterization was expressed as mean and standard deviation for continuous quantitative variables, percentage, and frequencies for categorical variables. The tests were also analyzed in a stratified way by distances from 180 to 240 km, 241 to 300 km, 301 to 360 km, and above 360 km. Events described in miles were converted to kilometers for categorization. To verify the distribution of the sample, when necessary, normality was verified using the Kolmogorov-Smirnov test, considering the total sample. The *t*-test for independent samples was applied to analyze the area under the curve. The ANOVA test (Two-Way) was used for PP and velocity comparisons between sex × time periods (years) and for velocity stratified between sex × distance, followed by the Bonferroni post hoc test. Partial eta square (*η*^2^) was calculated for each model and used as a measure of effect size considering small ≥ 0.01, medium ≥ 0.06, and large ≥ 0.14. The histogram of the age of male and female finalists was determined by 5-year age intervals. The relationship between velocity and distance was verified by Pearson's correlation. The magnitude of the correlation was determined as follows: *r* < 0.1, trivial; *r* = 0.1–0.3, small; *r* = 0.3–0.5, moderate; *r* = 0.5–0.7, strong; *r* = 0.7–0.9, very strong; *r* = 0.9–0.99, almost perfect; and *r* = 1.0, perfect. Statistical Package for Social Sciences (SPSS) version 20® and GraphPad Prism version 7.00, respectively, were used for all statistical and graphical analyses, adopting a significance level of *p* < 0.05.

## 3. Results

### 3.1. Continents and Countries' Ultramarathon Events

**Figure 1:**
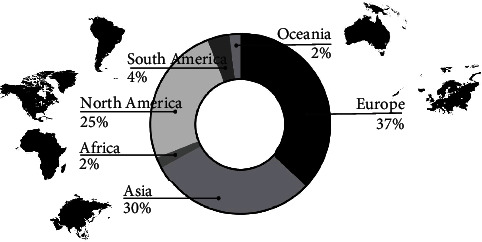
Distribution of ultramarathon events by continent.

**Figure 2:**
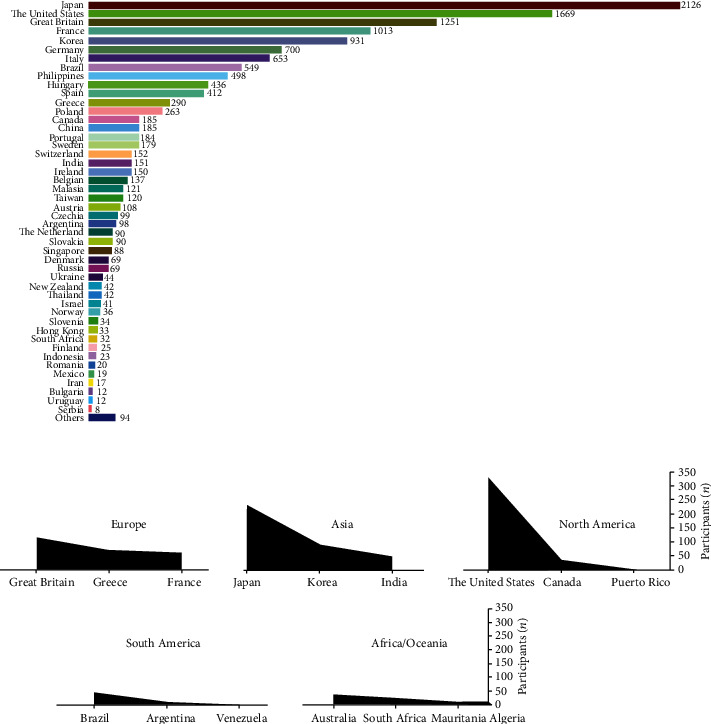
Distribution of ultramarathons by nationality and country events. (a) Number of competitors by nationality; (b) top three countries by continent that organized the most competitions.

Europe stood out with the largest number of organized events, followed by Asia and North America, totaling more than 92% of the UMs held in the world. The other continents had few (Figure 1). Japanese, American, British, French and Korean nationalities were the most common among UM runners (Figure 2(a)). The country with the highest absolute frequency (*n*_*i*_) of ultramarathon events was the Unites States (*n*_*i*_ = 329), followed by Japan (*n*_*i*_ = 231), Great Britain (*n*_*i*_ = 112), Korea (*n*_*i*_ = 91), and Greece (*n*_*i*_ = 70) (Figure 2(b)).

**Figure 3:**
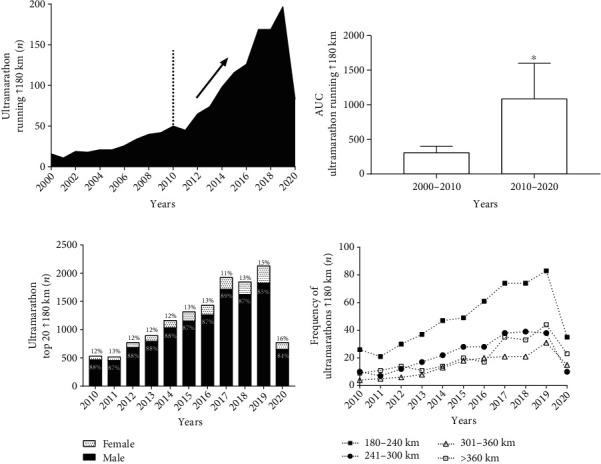
Analysis of the evolution of ultramarathons organized worldwide. (a) Number of ultraraces organized with distances over 180 km; (b) area under the curve; ^∗^*p* < 0.05; (c) percentage of finalists; (d) frequency of events organized over 180 km, stratified by distance.

Figure 3(a) shows an increase in the number of competitions from the year 2010, reaching a peak in the year 2019 with approximately 200 events. In 2020, the number of events was reduced to fewer than 100 events. There was an increase in the area under the curve in the decades from 2010 to 2020 when compared to the years 2000 to 2010 (1083 ± 517 vs.306 ± 93; *p* < 0.001) (Figure 3(b)). The number of women finalists in UMs in distances over 180 km represented a small percentage compared to men. An increase of 4% in female participation was observed in relation to the total number of participants, when comparing the years 2010 (12%) and 2020 (16%) (Figure 3(c)). Marathons with courses from 180 to 240 km were the most usual in all years. The frequency of UMs performed with distances above 360 km showed an increasing trend from the year 2016 when compared to distances from 301 to 360 km and in 2019 when compared to distances from 241 to 300 km (Figure 3(d)).

### 3.2. Sex Participation

When the absolute frequency of participation among men was verified, there was an increase of 405% between 2011 (*n*_*i*_ = 452) and 2019 (*n*_*i*_ = 1825), the years with the lowest and highest frequency of runners, while female participation increased 467% between 2010 (64) and 2019 (305) (Table 1).

### 3.3. Age of Peak Performance

**Figure 4:**
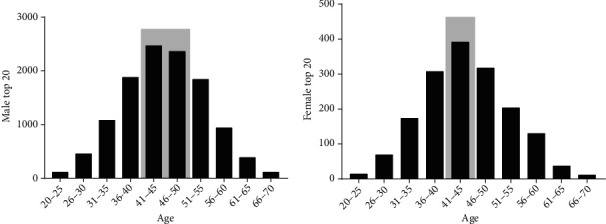
Histograms by age. (a) Histogram of the age of male finalists; (b) histogram of female age.

The distribution of male runners showed, in the years 2010 to 2020, between 41 and 50 years of age. This was five years older compared to the female distribution where runners were aged between 41 and 45 years. Participation in the top 20 ultramarathoners was less than 1% for runners aged 20 to 25 or over 65 years of age (Figures 4(a) and 4(b)).

**Figure 5:**
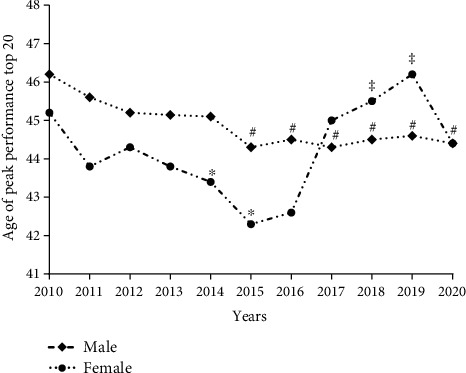
Age of peak performance. Legends: ^∗^female vs. male; ^#^men: 2010 vs. 2015, 2016, 2017, 2018, 2019, and 2020; ^‡^women: 2015 vs. 2018 and 2019. *p* < 0.05.

When analyzing the age of PP, the multifactorial model for performance showed significant results (*F* = 28.613, *p* < 0.001; *η*^2^ = 0.043), years (*F* = 27.388; *p* < 0.001; *η*^2^ = 0.020), and interactions: sex × years (*F* = 3.612, *p* < 0.001; *η*^2^ = 0.003). In contrast, there was no *difference* between sexes (*F* = 3.616, *p* = 0.057; *η*^2^ = 0.001). For men, there was a reduction in the years 2015 (44.3 ± 8.8; *p* = 0.005), 2016 (44.4 ± 8.5; *p* = 0.011), 2017 (44.3 ± 8.5; *p* = 0.002), 2018 (44.5 ± 8.6; *p* = 0.010), 2019 (44.5 ± 8.6; *p* = 0.016), and 2020 (44.4 ± 8.3; *p* = 0.044) when compared to 2010 (46.1 ± 8.9). Women showed an increase in age in 2019 (46.2 ± 9.4) when compared to 2015 (42.3 ± 8.3; *p* < 0.001) and 2016 (42.5 ± 7.9; *p* = 0.001) and in the year 2018 (45.5 ± 8.3) to 2015 (42.3 ± 8.3; *p* = 0.0230). When there was a difference between the sexes, women were younger in 2014 (43.4 ± 8.5 vs.45.0 ± 8.8; *p* = 0.030), 2015 (42.3 ± 8.3 vs.44.3 ± 8.8; *p* = 0.006), and 2016 (42.5 ± 7.9 vs.44.4 ± 8.5; *p* = 0.006) (Figure 5).

### 3.4. Performance

**Figure 6:**
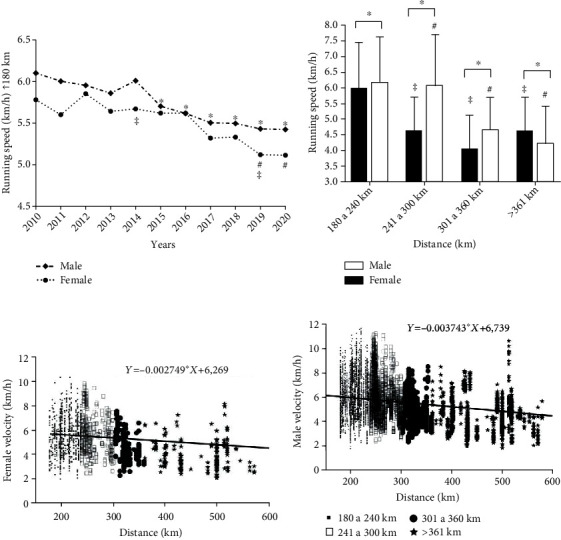
Results of performance comparison between the sexes and distance. (a) Average velocity. ^∗^Male 2010 vs. 2015, 2016, 2017, 2018, 2019, and 2020; ^#^women 2012 vs. 2019 and 2020; ^‡^2014 and 2019. Female vs. male. *p* < 0.05. (b) Average velocity stratified by different distances; ^∗^male vs. female; *p* < 0.001. ^#^Female 180 to 240 km vs. 241 to 300 km, 301 to 360 km, and >360 km; *p* < 0.001. ^‡^Male 180 at 240 km vs. 241 at 300 km, 301 at 360 km, and >360km; *p* < 0.001. (c) Velocity correlation stratified by different distances for women; (d) velocity correlation stratified by different distances for men.

When analyzing average velocity over the years, the multifactorial model for performance showed significant results (*F* = 14.435, *p* < 0.001; *η*^2^ = 0.021), for sex (*F* = 22.722, *p* < 0.001; *η*^2^ = 0.002) and years (*F* = 11.712; *p* < 0.001; *η*^2^ = 0.008), but no relationships between sex × years (*F* = 0.769, *p* = 0.659; *η*^2^ = 0.001).  Figure 6(a) shows a reduction in average velocity over the years for male runners from 2015 (5.7 ± 1.5 km/h; *p* < 0.001), 2016 (5.6 ± 1.5 km/h; *p* < 0.001), 2017 (5.5 ± 1.5 km/h; *p* < 0.001), 2018 (5.4 ± 1.6 km/h; *p* < 0.001), 2019 (5.4 ± 1.6 km/h; *p* < 0.001), and 2020 (5.4 ± 1.6 km/h; *p* < 0.001) when compared to the year 2010 (6.1 ± 1.7 km/h). As for women, there was a reduction in the average velocity in 2019 (5.1 ± 1.5 km/h; *p* < 0.001) and 2020 (5.1 ± 1.7 km/h; *p* < 0.001) when compared to 2012 (5.8 ± 1.7 km/h). When comparing the average velocity between the sexes, women presented a lower speed than men in 2014 and 2019 (*p* < 0.001). In the analysis stratified by distance (Figure 6(b)), women had lower mean speed than men in all stratifications (*p* < 0.001).

When analyzing average velocity stratified by different distances, the multifactorial model for performance showed significant relationships (*F* = 8.612, *p* < 0.001; *η*^2^ = 0.195), for sex (*F* = 7.495, *p* = 0.006; *η*^2^ = 0.001) and distance (*F* = 31.920; *p* < 0.001; *η*^2^ = 0.334) but no relationship between sex × distance (*F* = 0.871, *p* = 0.881; *η*^2^ = 0.010). Men and women showed higher velocity in distances from 180 to 240 km (6.14 ± 1.47 km/h vs.5.97 ± 1.48 km/h) when compared to 241 to 300 km (6.0 ± 1.64 km/h vs.4.59 ± 1.09 km/h; *p* < 0.001), 301 to 360 km (4.6 ± 1.05 km/h vs.4.0 ± 1.08 km/h; *p* < 0.001), and >360 km (4.1 ± 1.20 km/h vs.4.5 ± 1.09 km/h; *p* < 0.001), respectively. A negative linear regression (Figures 6(c) and 6(d)) was observed for the velocity of women (*Y* = −0.002749∗*X* + 6.269; *r* = −0.3; *p* < 0.001) and men (*Y* = −0.003743∗*X* + 6.739; *r* = −0.3; *p* < 0.001) with increasing distance.

**Figure 7:**
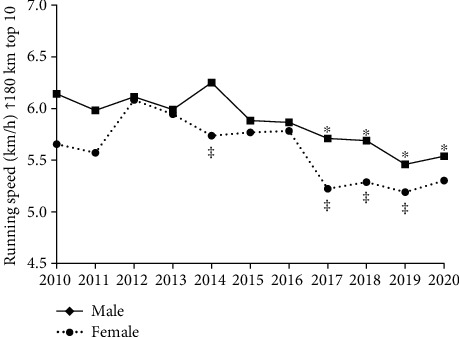
Running top 10 finalist speeds: ^∗^male 2010 vs. 2017, 2018, 2019, and 2020; ^‡^female vs. male 2014, 2017, 2018, and 2019.

Also, when analyzing the top 10 average velocities over the years, the multifactorial model for performance showed significant effects (*F* = 10.442, *p* < 0.001), for sex (*F* = 20.149, *p* < 0.001) and years (*F* = 8.600, *p* < 0.001), but no relationships between sex × years (*F* = 0.885, *p* = 0.546).  Figure 7 shows no reduction in the average velocity over the years for female runners. When comparing the average velocity between sexes, women presented a lower speed than men in 2014 (*p* = 0.008), 2017 (*p* = 0.002), 2018 (*p* = 0.008) and 2019 (*p* = 0.038). Men reduced average velocity from 2017 (5.7 ± 1.5 km/h; *p* = 0.003), 2018 (5.6 ± 1.6 km/h; *p* = 0.001), 2019 (5.4 ± 1.8 km/h; *p* < 0.001), and 2020 (5.5 ± 1.6 km/h; *p* < 0.001) when compared to the year 2010 (6.1 ± 1.9 km/h).

## 4. Discussion

The aim of the present study was to analyze the age peak performance progression (PP) and ultramarathon running (UMs) events performed over 180 km. We analyzed 1202 races from the last two decades (2000 to 2020) and individual data from 13,300 runners between the years 2010 to 2020. The results show the following: (i) The continent of Europe hosted the highest number of events in the world; (ii) Japanese nationals were the most common among UMs runners. (iii) The United States stood out as the country that had the most organized UM competitions. (iv) Marathon runs between 180 and 240 km were the most recurrent, especially after 2016, when there was an advance in the number of races over 360 km, run at a lower speed. (v) The number of women finalists in UMs represented a low percentage compared to men. (vi) Men accounted for more than 80% of the runners, showing a reduction in PP from 2015 onwards. (vii) The distribution of ages by age group showed a male PP from 41 to 50 and female from 41 to 45. The concentration in PP age in both sexes occurred on average at 45 years of age.

When we look at the number of competitions organized during the period 2000 to the year 2020, a significant advance was noticed from the year 2010, reaching almost 200 competitions in the year 2019. A drop was also observed in the year 2020, explained by the COVID-19 pandemic, which caused, in addition to the cancellation of major sporting events such as the Tokyo Olympics and the Euro Cup, as well as cancellation of the UMs competitions. Naturally, the restrictive measures adopted by each country, and the closed borders justified the reduction in the number of organized events [11].

### 4.1. Continent

The highest number of ultramarathoners around the world came from Europe, followed by Asia and North America. Although, when analyzing the country of nationality of the participating athletes, The United States and Japan were the countries with the highest number, the European continent, in addition, hosted the great traditional world competitions, especially in the United Kingdom, France, Germany, and Italy (e.g., Sandstone Way Ultra 200 Km (GBR); Jurasteig Nonstop Ultratrail 230 km (GER); Paris-Colmar (FRA); Tor des Géants—330 km Endurance Trail della Valle d'Aosta (ITA)). This information corroborates the study by Silva [11] highlighting an increase in American, Japanese, German, Italian, and Polish participants in 100 km races over the last 14 years. Japanese runners were highlighted at times for sprint running. The finding that runners from Japan were among the best in the 10 km, half-marathon, marathon, and 100 km ultramarathon running has been previously reported [8]; Japanese participation continued advancing and, also, the number of large, organized UMs. In contrast, Africa, although it is the continent with the highest number of countries and has had the greatest success of East African athletes in long-distance running over the past 40 years (e.g., Kenyan and Ethiopian endurance runners) [12, 13], presented a low number of UM competitions.

When evaluating the quantity of the UM events in a stratified way, a highlight was noted for the courses between 180 and 240 km. Competitions with these specific distances were the most organized, possibly because they had more favorable logistics: these distances can be covered in 1 or 2 days, making them less complex than events organized over multiple days. On the other hand, interestingly, competitions over 360 km began to become common in 2017, a fact that should be further explored. In part, it can be explained by the increase in psychological resilience [9].

### 4.2. Sex Participation

Males were predominant among the top 20 ultramarathoners in the UMs in all evaluated years. Female representation was also low in events with courses closer to 100 km [14]. However, in marathons, female participation has increased, with proportions similar to males, even in the younger age groups [2]. On the other hand, we can observe over the years that there has been a slight evolution in the number of women runners at UMs, suggesting a possible reduction in the difference in the *speed* of men/women, perhaps explained by cultural changes, social and sporting scenarios favoring female engagement in sport [15].

### 4.3. Age of Peak Performance

Interestingly, from 2017 onwards, the average age of female participants in the UMs increased by 3 years when compared to 2015. In contrast, from 2015 onwards, the average age of the men decreased by practically 2 years when compared to 2010. In recent years, parallel to the development of training and nutritional variables, ultraendurance running has experienced exponential growth in popularity, with more organized events each year [16]. We believe that competitions equal to or greater than 180 km have accompanied this popularity as well as the tendency towards reduction in the average age of the participants. In addition, many young runners under the age of 19 are participating in 100-km events, especially over the last couple of decades [17].

In general, we observed that the age of PP in 180+ km runs averaged 45 years, 5 years older than 100 km runners [2], 10 years older than the marathon runners, and 20 years older than half-marathon runners [4]. The highest age of peak performance in ultramarathoners compared to that in half-marathoners and marathoners is related to the fact that most of the time, the ultramarathoners have completed many marathon races before participating in a UMs. On the other hand, age of PP found in runs of 180 km and over was similar to what was observed in races of 100 km [2].

Most male participants were in the range of 41 to 50 years old, being older than women by 5 years. Women had a shorter age range, between 41 and 45 years, possibly justified by the fact of greater male representation. However, the PP of men by age was higher than that of women only in 2014, 2015, and 2016. These results are also in agreement with the findings of Romer et al. [18], identifying the average age in the 200 km races for both sexes close to 42 years of age.

### 4.4. Ultramarathoners' Performance

There was a reduction in running speed among men in the years 2015 to 2020. This trend, although similar for the women runners, as verified by linear regression, was only actually true for the years 2019 and 2020. This reduction can be related to the increase in the number of participants in events with distances above 360 km and average speeds around 4 to 4.5 km/h. As verified by Hoffman et al. [19], the increase in number of runners was not associated with an increase in number of events. These results are explained by changes in the profile of the runners, given that most of them were recreational runners, with objectives of improving their health, quality of life, and not necessarily their performance [20, 21].

An analysis of the top 10 ultramarathoner runners showed that women kept at the same speed over the years. That is, although there was a substantial difference between the top 20 and the top 10 finalists, when comparing running speed, only the first 10 finalists manifested changes in performance pattern. Another fact can also be observed with the increase in the differences between male and female. Significant differences in running performance between the sexes were shown only in the 2014 and 2019 events. Among the top 10 ultramarathoners, the difference in running performance was greater in 2014, 2017, 2018, and 2019, with male athletes doing better than female athlete.

In general, sex differences in endurance sports were ≈12% [22]. A plethora of factors can be associated with performance differences, i.e., anthropometric characteristics, physiological indicators, and training commitment [6, 7]. However, in recent studies woman have reduced the performance difference [23]. Another aspect is the decrease in the physiological index associated with endurance performance. For example, the maximum oxygen uptake (VO_2_max) or cardiac output decreases with increasing age [24]. Besides this, changes in training commitment, nutritional habits, and body composition alterations can explain these results.

### 4.5. Limitations and Future

New studies should include more characteristics of runners such as aerobic capacity, muscle mass, fat mass, and experience of training. Although the DUV research source presents a large amount of data and makes it an easy way to check out the global UMs, the data is retrospective. In order to expand the analysis of the performance progression, a comparison with other data and not just with the top 20 finalists is suggested. The stratification of events according to different climatic conditions should be investigated. On the other hand, to our knowledge, this is the first study that has analyzed participation, age peak, and performance progression in UMs with distances longer than 180 km.

### 4.6. Practical Implications

Although Ethiopia and Kenya runners were the most numerous in the 10 km, half-marathon, and marathon runs, Africans were underrepresented in the UMs races. The number of Japanese ultramarathoners increased. Women had limited representation in the UMs. Men were the fastest in all distance events. This information is of great practical value for coaches who work with distance runners. Being aware of the role that participation, age peak and performance progression can help coaches design exercise programs and make decisions about the most suitable running distance for their trainees. Adapted medical assessment for the older athletes could be useful in avoiding medical events such as sudden cardiac death. For coaches and older athletes preparing for an ultramarathon, specific training plans and nutrition plans for the elderly athletes could be designed.

## 5. Conclusions

The past decade has seen an increase in the number of ultramarathon running events. The European continent had the highest number, followed by Asia and North America. There was an exponential increase in the participation of men, with a much lower participation of women. The age peak performance to the world's, top 20 finalists, was on average 45 years. Men and women showed higher velocity in distances from 180 to 240 km when compared to 241 to 300 km, 301 to 360 km, and >360 km. The average running speed reduced over the years; this fact is associated with an increase in the number of participants in the events and not specifically related to a decline in athletic performance.

## Figures and Tables

**Table 1:** Frequency of participation by sex in the top 20 ultramarathons ≥ 180 km, 2010-2021.

Year	Male			Female			Total		
	*n*_*i*_	*N*_*i*_	*F*_*i*_ (%)	*n*_*i*_	*N*_*i*_	*F*_*i*_ (%)	*n*_*i*_	*N*_*i*_	*F*_*i*_ (%)
2010-2011	467	467	4.0	64	64	3.9	531	531	3.9
2011-2012	452	919	7.9	66	130	7.9	518	1.049	7.8
2012-2013	684	1.603	13.8	87	217	13.1	771	1.820	13.6
2013-2014	792	2.395	20.6	104	321	19.4	896	2.716	20.4
2014-2015	1.033	3.428	29.4	131	452	27.3	1.640	3.880	29.1
2015-2016	1.149	4.577	39.3	168	620	37.5	1.317	5.197	39.0
2016-2017	1.261	5.838	50.1	172	792	47.9	1.433	6.630	49.8
2017-2018	1.712	7.550	64.8	211	1.003	60.6	1.923	8.553	64.3
2018-2019	1.620	9.170	78.7	226	1.229	74.3	1.846	10.399	78.1
2019-2020	1.825	10.995	94.4	305	1.534	92.7	2.130	12.529	94.2
2020-2021	651	11.646	100.0	120	1.654	100.0	771	13.300	100.0

*n*_*i*_: absolute frequency; *N*_*i*_: accumulated frequency; *F*_*i*_: accumulated relative frequency.

## Data Availability

The datasets used and/or analyzed during the current study are available from the corresponding author on reasonable request.
